# Detection of Atmospheric Water Deposits in Porous Media Using the TDR Technique

**DOI:** 10.3390/s150408464

**Published:** 2015-04-13

**Authors:** Anna Nakonieczna, Marcin Kafarski, Andrzej Wilczek, Agnieszka Szypłowska, Grzegorz Janik, Małgorzata Albert, Wojciech Skierucha

**Affiliations:** 1 Laboratory of Dielectric Spectroscopy, The Bohdan Dobrzański Institute of Agrophysics of the Polish Academy of Sciences, ul. Dos´wiadczalna 4, Lublin 20-290, Poland; E-Mails: m.kafarski@ipan.lublin.pl (M.K.); a.wilczek@ipan.lublin.pl (A.W.); a.szyplowska@ipan.lublin.pl (A.S.); w.skierucha@ipan.lublin.pl (W.S.); 2 Institute of Environmental Protection and Development, Wrocław University of Environmental and Life Sciences, Pl. Grunwaldzki 24, Wrocław 50-363, Poland; E-Mails: grzegorz.janik@up.wroc.pl (G.J.); malgorzata.albert@up.wroc.pl (M.A.)

**Keywords:** atmospheric water deposits, time-domain reflectometry, porous materials

## Abstract

Investigating the intensity of atmospheric water deposition and its diurnal distribution is essential from the ecological perspective, especially regarding dry geographic regions. It is also important in the context of monitoring the amount of moisture present within building materials in order to protect them from excessive humidity. The objective of this study was to test a constructed sensor and determine whether it could detect and track changes in the intensity of atmospheric water deposition. An operating principle of the device is based on the time-domain reflectometry technique. Two sensors of different plate volumes were manufactured. They were calibrated at several temperatures and tested during field measurements. The calibration turned out to be temperature independent. The outdoor measurements indicated that the upper limits of the measurement ranges of the sensors depended on the volumes of the plates and were equal to 1.2 and 2.8 mm H_2_O. The respective sensitivities were equal to 3.2 × 10^−3^ and 7.5 × 10^−3^ g·ps^−1^. The conducted experiments showed that the construction of the designed device and the time-domain reflectometry technique were appropriate for detecting and tracing the dynamics of atmospheric water deposition. The obtained outcomes were also collated with the readings taken in an actual soil sample. For this purpose, an open container sensor, which allows investigating atmospheric water deposition in soil, was manufactured. It turned out that the readings taken by the porous ceramic plate sensor reflected the outcomes of the measurements performed in a soil sample.

## 1. Introduction

In arid and semi-arid regions, the amount of water supplied to the soil due to the existence of fog, dew, hoarfrost and direct water adsorption from the atmosphere can exceed that of rainfall [1–3]. For this reason, the atmospheric water deposits can be the main source of liquid water for plants and other living organisms in continuously or temporarily dry ecosystems [4–7]. In dry regions, the fog harvesting technique was employed to pull the water out of the humid air that drifts from nearby oceans [8–10]. It allows one to supply significant quantities of potable water in places where rainfall is rare. The knowledge of the amount of water uptake by the soil in the form of atmospheric water deposits enables one to select the plants adapted to the specific moisture conditions. This is particularly important in areas where soils are prone to water and wind erosion, because the properly established plant cover facilitates preventing their further degradation [11]. On this account, the research concerning atmospheric water deposition is of crucial importance from the ecological perspective. The two main aspects of its investigations are the measurements of the amount of water added to the soil and examining the diurnal distribution of the atmospheric water deposition intensity. The intensity of the discussed phenomenon is the amount of deposited water per a selected time period. It is related to the dynamics of the formation and evaporation of atmospheric water deposits.

Additionally to soil, which is a porous medium of complex composition and pore distribution, the possibility of estimating the dynamics and intensity of water deposition is also important for other porous media, such as various building materials. It is essential for monitoring the moisture of monuments and construction elements of buildings in order to protect them from the deleterious effects of humidity [12]. Among methods used for this purpose, two main approaches can be distinguished. They are based on investigations of diversified electromagnetic [12,13] or thermophysical [14] phenomena related to the existence of water in gaseous, liquid or solid states in the porous medium.

Several devices and methods were proposed for measuring the intensity of the atmospheric water deposition. Duvdevani blocks [15], the cloth plate method [16], the Hiltner dew balance [17] and the dew recorder of the Kessler-fuel-type [18] rely on estimating the amount of water deposited on various non-porous artificial surfaces. The first two of the above methods are based on a manual collection of readings. The measurements reflect the total water deposition per a specific period of time that cannot be arbitrarily small due to limitations in human perception. Hence, satisfactorily precise minute readings are impossible and, thus, the results cannot be used for estimating the diurnal distribution of atmospheric water deposition intensity [3]. The measurements carried out by the remaining two gauges are automated, and hence, they enable continuous measurements of the mass of deposited water. Several extended versions of the devices, whose sensing element is the plate, on which the atmospheric water deposition occurs, were proposed [19,20]. They allow for automated measurements and are successfully used in tracing the intensity of deposited water [21], as well as in collecting water samples from atmospheric moisture [19].

The thermal properties of the condensation plates and, thus, the amount of water deposited on them differ significantly in comparison to surfaces, such as soil or other porous media. Moreover, their use does not allow for a reliable estimate of the water adsorption effect in porous materials due to dissimilarities in the bulk structure, mainly the significantly smaller specific surface area. For the above reasons, the volume of water deposits estimated with the use of Duvdevani blocks and the mass of deposited water measured with the remaining above devices cannot be interpreted as absolute. The obtained values may only serve for comparison between different sites and time periods [6,22].

Another group of devices that measure the atmospheric water deposition intensity consists of the electrical conductance soil-moisture meter [23] and (micro-)lysimeters [11,24–27]. These devices are often large, and hence, their deployment is problematic. The readings are taken in actual soil samples, so the effect of water adsorption on the solid phase boundary is taken into account. The principle of the former gauge operation is measuring the changes in soil electrical conductance due to the atmospheric water deposition. The measurement affects the heat transport within the soil and disturbs the structure of the material [3]. In the case of lysimeters, the measured mass of deposited water is usually equal to or less than the device uncertainty [24,28], and this drawback has been only partially eliminated [11]. A device that is an interesting combination of a plate measurer and a lysimeter was proposed recently [29]. It has satisfactory sensitivity and resolution for measuring atmospheric water deposition. It also enables taking measurements using various canopy types.

Thorough and informative reviews of the methods employed in measuring atmospheric water deposition along with a rich literature on the subject were presented in [3,29,30]. Although many diversified devices and methods for measuring the atmospheric water input into the soil, as well as other porous media were proposed, the international standard was not hitherto established. This is mainly due to the obstacles to measuring small water additions to the medium, problems with taking readings in actual samples and atmospheric disturbances during their course. Therefore, there still exists the need for automated devices, which allow for the correct estimation of the atmospheric water deposits uptake and whose resolution is high, because the intensity of the atmospheric water deposition is usually very low.

The objective of the current study was two-fold. The first was to construct a sensor for the atmospheric water deposits measurements, which would allow for automated readings that, in the case of outdoor measurements, could be collected remotely. The design of an innovative sensor with a condensation element in the form of a porous ceramic plate, whose operating principle is based on the time-domain reflectometry (TDR) technique, is presented in the first part of the paper. The construction of the sensor is the subject of the patent application [31]. The following research goal was to test the sensor for the detection and tracking the changes in intensity of the atmospheric water deposition. Field measurements were performed in order to fulfill this task. The outcomes of an outdoor experiment are presented in the final part of the paper, which is preceded by the calibration of the device. The readings taken by the developed sensor were also collated with the measurements performed in an actual soil sample. A device analogous to the porous ceramic plate sensor, which allows taking measurements in soil, was fabricated in order to achieve the goal.

## 2. Materials and Methods

### 2.1. Porous Ceramic Plate Sensor Construction

**Figure 1:**
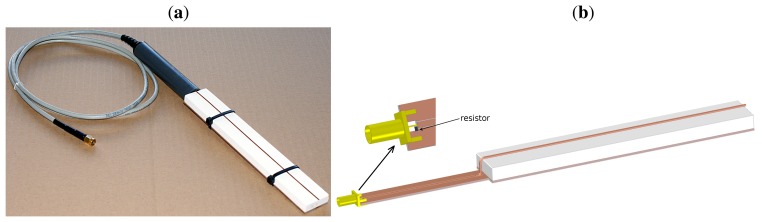
(**a**) The porous ceramic plate sensor for atmospheric water deposits measurements; (**b**) a scheme of the device.

The construction of the sensor designed for the atmospheric water deposition measurements is presented in Figure 1. The functional element, which served as a water collector, was a plate made of corundum ceramics. The plate was placed between a flat surface made of epoxy laminate covered with copper, which was impermeable to water, and a copper wire. The metallic layer and the wire formed a parallel transmission line untypical for a TDR probe, in which the bottom surface was the mass and the wire was the signal electrode. Both elements of the transmission line closely adhered to the ceramic plate in order to eliminate the formation of air gaps, what enhanced the accuracy of the measurements.

Two sensors with a ceramic plate thickness equal to 4.1 and 8.7 mm were produced. From now on, the devices will be referred to as Sensors 1 and 2, respectively. The length and width of each ceramic plate were equal to 19.2 and 2.9 cm, respectively. The length was adjusted to technical requirements of the detecting device when operating in the moisture range of interest [32]. The width of the porous plate was selected so that the plate encompassed the whole sensor sensitivity zone below the wire. The area of the top surface of both ceramic plates was equal to 55.7 cm^2^. The volumes of the plates were equal to 22.8 and 48.4 cm^3^ for Sensors 1 and 2, respectively. The research was conducted with the use of two sensors of different thicknesses of porous ceramic plates in order to check whether the readings may be correlated either with the area or volume of the plate.

The sintering temperature applied during the ceramics fabrication was equal to 1250 °C, and the final material porosity was about 25%. Such ceramics was successfully used also for manufacturing soil tensiometers, because of its very good mechanical properties in comparison to other porous materials [33]. The spatial internal structure of the corundum ceramics is highly reproducible. The distribution of pore volumes and the specific surface area depend on the sintering temperature and, to a lesser extent, on the technology of the molding compound preparation. The properties of the porous plates were insensitive to changeable atmospheric conditions. They were the same for all plates produced at a specific temperature, and they did not change in time. This ensured high measurement repeatability, not only for a particular device, but for all sensors with ceramic plates fabricated at the same sintering temperature. Moreover, the results were obtained immediately due to rapid and uniform atmospheric water absorption by the material's capillaries. Hence, the dependence of the measurement results on environmental (especially wind) conditions was minimized in comparison to non-porous condensation elements. The porosity of the material of the plates allowed taking the effect of direct water adsorption on the phase boundary into account more accurately than in the case of non-porous deposition elements.

### 2.2. Open Container Sensor Construction

**Figure 2:**
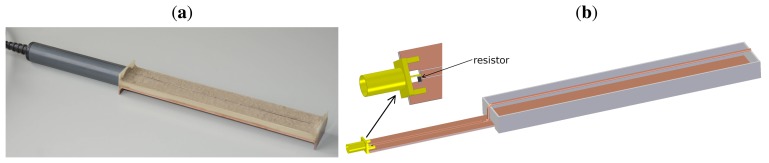
(**a**) The sensor for measurements of the amount of atmospheric water deposited in a soil sample; (**b**) a scheme of the device.

In order to perform comparative analyses, a device that allows making measurements in soil samples was manufactured. The construction of an open container sensor, which was analogous to Sensor 1 described above, is shown in Figure 2.

The base made of epoxy laminate covered with copper served as the bottom of a cuboidal open container into which the soil was placed. On the top of the soil sample, a copper signal wire was placed. The dimensions of the interior of the cuboidal container were the same as the dimensions of the porous ceramic plate of Sensor 1. It allowed comparison between the readings taken with the two devices.

### 2.3. Principle of Operation of the Sensors

The operating principle of the designed probes was based on the TDR technique [34]. The time of the signal propagation (the TDR time) was calculated as an interval between a passage of the forward and return signals through a 240-ohm resistor, which served as a marker. It was positioned at the beginning of the probe, as depicted in Figures 1 and 2. The TDR time depended on the bulk dielectric permittivity of the functional element of the sensor. The bulk dielectric permittivity was, in turn, related to the amount of liquid water present within the porous material. The obtained results were not affected by other factors, such as ions dissolved in the deposited water, dust or other contaminants, which could appear within the sensitivity zone during outdoor measurements. This fact made the TDR technique highly accurate for atmospheric water deposition measurements. Moreover, the technique allowed conducting continuous experiments due to automation and multiplexing feasibility, which was a considerable advantage in the planned atmospheric water deposition measurements. The employed method also enabled taking readings at frequent intervals, which, in combination with high measurement precision, provided an opportunity to investigate the long-term dynamics of the intensity of atmospheric water deposition in a highly accurate way.

The designed sensors worked in tandem with the low-energy eight-channel integrated measuring module TDR/MUX (manufacturer: E-Test Ltd., Lublin, Poland), which is a needle pulse signal analyzing TDR meter [35]. The device was connected to the power supplier and ensured the signal generation and the TDR time readings.

The amount of liquid water present in the bulk of the ceramic plate in particular ambient conditions, which is detected by the sensor, depends crucially on numerous properties of porous materials. The important characteristics can be divided into two groups, which are related to the chemical composition of the solid material and its spatial structure [3,36]. The former class contains, e.g., interaction energies on the phase boundary and wettability, which determines the hydrophobic/hydrophilic character of the medium and is described by the contact angle on the solid-liquid interface. The latter group of properties includes, e.g., the specific surface area of the material, its porosity and pore size distribution, as well as the swelling phenomenon, which is characteristic for moist soil.

The outlined properties affect the phenomena and processes that regulate the gas-liquid-solid state equilibrium, in which water remains within the porous material in the existing temperature and pressure conditions. Among the phenomena and processes, water adsorption, vapor pressure within the pores, diffusion and transport of both gaseous and liquid water through the porous medium or capillary phenomena can be listed.

The properties of the corundum ceramics, which determine the amount of water deposited within it, can be characterized in the laboratory. These features are controllable, and they do not change in time, because the chemical composition and spatial structure of the porous material are stable. For this reason, the developed sensor can be potentially used as a reference for investigations of the aforementioned phenomena and processes in various types of soils, as well as other porous materials.

### 2.4. Temperature Dependence of the Sensor Performance

As a preliminary analysis of the produced devices, the TDR measurements of dry porous ceramic plates were conducted at various temperatures varying from 5 to 30 °C. This allowed testing the influence of temperature on the dielectric properties of the porous material itself.

The porous ceramic plate sensor was intended to work at low moisture due to the characteristic of the investigated phenomenon, *i.e.*, the atmospheric water deposition. For this reason, the dependence of the dielectric permittivity of the ceramic plate-water system and, hence, the measured TDR time on temperature could be neglected [37]. In order to confirm the statement, the calibration was performed for both probes in a wide temperature range. Six temperature values equal to 5, 10, 15, 20, 25 and 30 °C were chosen for the calibration.

After saturating the ceramic plate with water, each of the sensors separately was placed on a scale and connected to the TDR meter. Both measuring devices were controlled by a custom written PC application with a data logger function. During water evaporation from the porous material, automated readings of mass and TDR time were collected simultaneously every minute. Thus, for each of the probes, the TDR time was correlated with the water content of the medium. During the readings, the sensor placed on the scale was kept in a thermally-stable environment ensured by a climatic chamber WKL 100 (manufacturer: Weiss Technik, Berlin, Germany), within which the humidity was equal to 50%. The scale was placed on an anti-vibrational pad in order to enhance the stability of the readings.

### 2.5. Outdoor Measurement Setup

**Figure 3:**
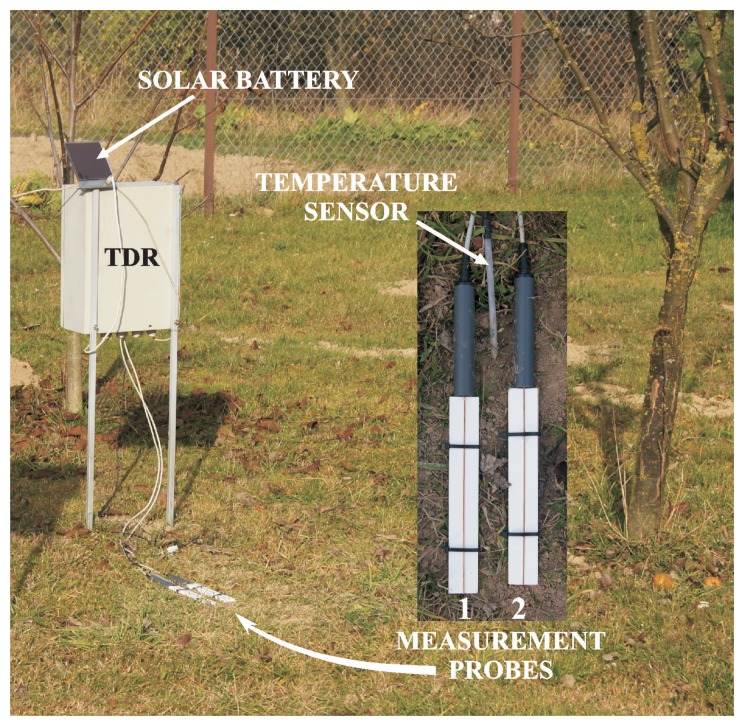
The measurement station in Stasin, Lublin Voivodeship, Poland.

The performance of the produced sensors was tested during field measurements. The probes connected to the TDR/MUX measuring unit supplied by the solar cell were put in the measurement station, which was placed in eastern Poland (Stasin, Lublin Voivodeship). It is shown in Figure 3. The detailed description of the construction and capabilities of the measurement station was presented in [38]. In the current setup, the FP/mts two-rod probe for soil moisture, electrical conductivity and temperature readings was substituted by the porous ceramic plate and open container sensors, which were integrated with the station. The temperature sensor was an element of the measurement system and was connected to the AUX input of the TDR/MUX.

Together with the field measurements conducted with the two porous ceramic plate sensors, outdoor readings in an actual soil sample were taken. For this purpose, a device, shown in Figure 2, was used. It was also integrated with the measurement station, so that it could be used instead of the FP/mts probe.

### 2.6. Properties of the Examined Soil

The soil sample used for the experiment was at first characterized. The particle size distribution was examined using the laser diffractometer Mastersizer 2000 (manufacturer: Malvern Instruments, Malvern, UK) with the Hydro G dispersion unit. The particles from 0.01 µm to 2 mm in diameter were measured. The following device parameters were set: a stirrer speed equal to 700 r.p.m. and a pump speed of 1750 r.p.m. [39]. The Mie theory with the refraction index and the absorption coefficient for the dispersed phase equal to 1.52 and 0.1, respectively, was used. The refraction index for the continuous phase was set equal to 1.33. Each measurement was repeated three times for three independent dosages of the soil. A single measurement lasted one minute, that is 30 s of red and 30 s of blue light exposure [40]. The percentage particle size distribution of the examined soil is presented in Table 1.

## 3. Results and Discussion

### 3.1. Sensor Calibration

The introductory measurements of dry porous ceramic plates conducted at several temperatures varying from 5 to 30 °C revealed that the temperature did not influence the dielectric properties of the dry material, and hence, it did not affect the signal propagation. This was a considerable advantage of the ceramics used for manufacturing the functional element of the sensor, because the device was sensitive only to the amount of water deposited within it.

The amount of water that was needed for the saturation of the ceramic plate was equal to 6.50 and 15.75 g for Sensors 1 and 2, respectively. It was controlled during measurements at all temperatures. The mass of water determined the upper limit of the measurement range of each of the devices. The limit was equal to 0.12 g·cm^−2^, that is 1.2 mm H_2_O, for Sensor 1 and 0.28 g·cm^−2^, *i.e.*, 2.8 mm H_2_O, for Sensor 2.

**Figure 4:**
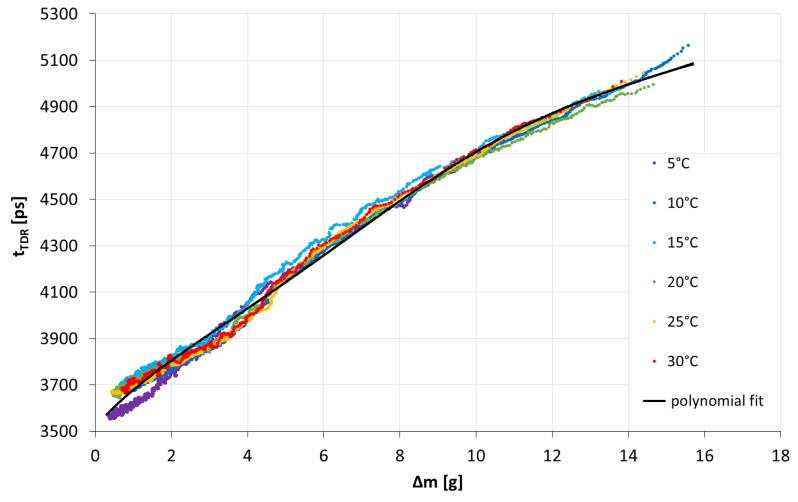
The calibration of Sensor 2 at temperatures ranging from 5 to 30 °C, *i.e.*, the TDR time, *t_TDR_*, as a function of the amount of water present in the porous plate, Δ*m*, which, for the dry plate, is equal to zero.

The calibration curves depict the TDR time as a function of the water amount in the porous plate. Since their shapes were similar for both tested devices, the curve obtained for Sensor 2 is shown in Figure 4 by way of illustration. The data on the graph represent the moving average of experimental points, which was calculated out of eleven point sets. This allowed reducing the amount of displayed points in order to enhance the clarity of presentation. The slight scattering of experimental points was mainly due to uncertainties in the mass measurements. They resulted from the fact that the scale was placed in a climatic chamber, which trembled slightly while working. However, due to frequent readings and, hence, a large amount of data, random deviations did not influence the final outcome, which was the calibration curve.

As may be inferred from Figure 4, the calibration did not depend on temperature, and hence, a single calibration curve was needed. This fact facilitated using the sensor at temperatures from outside of the range used during calibration provided that water remained in a liquid state in ambient atmospheric conditions. The best fit to the experimental data was a fourth order polynomial with *R*^2^ equal to 0.9876 and 0.9905 for Sensors 1 and 2, respectively. It was fitted to the data in order to obtain the equation of the calibration curve. Two calibration curves Δ*m* = *f* (*t_TDR_*) were obtained, one for each sensor. The root mean square errors related to both calibration curves for all considered temperatures are presented in Table 2.

The RMSE values indicated a similar quality of the fit for all temperatures and for the whole temperature range. The overall analysis supported the above statements that a single calibration curve for a particular sensor was sufficient and that the scattering of experimental points did not influence the calibration undesirably.

### 3.2. Field Measurements

**Figure 5:**
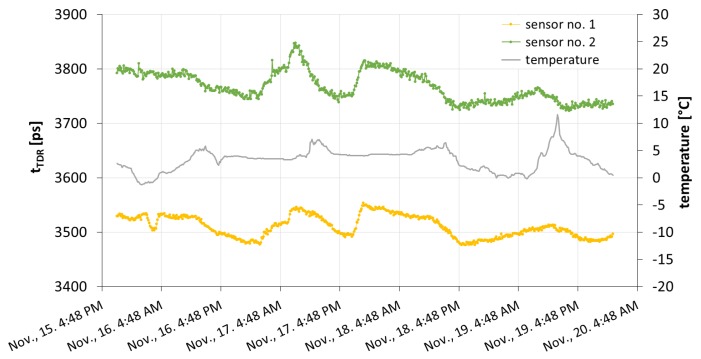
The TDR time, *t_TDR_*, recorded by both sensors and accompanying temperature readings collected in November, 2013.

The outdoor readings collected in November 2013, by the TDR meter equipped with the temperature sensor are shown in Figure 5. The presented time period was chosen, such that it did not include rainy weather, because the tested devices were devoted to measuring small water additions to the medium, which are characteristic for atmospheric water deposits.

The values of TDR time recorded by both devices differed due to different amounts of water within the sensor sensitivity zones. Although the TDR time values were higher for the sensor with a thicker porous plate, the tendencies in both cases were the same. During the experiment, there were two time periods with approximately constant temperature, *i.e.*, the nights of 16 to 17 and 17 to 18 November, approximately from 5 p.m. to 7 a.m. Due to the fact that despite the constant temperature, the TDR time readings were varying, it is clear that the measurements were not affected by the temperature, and the sensors possess an ability to detect the atmospheric water deposits.

**Figure 6:**
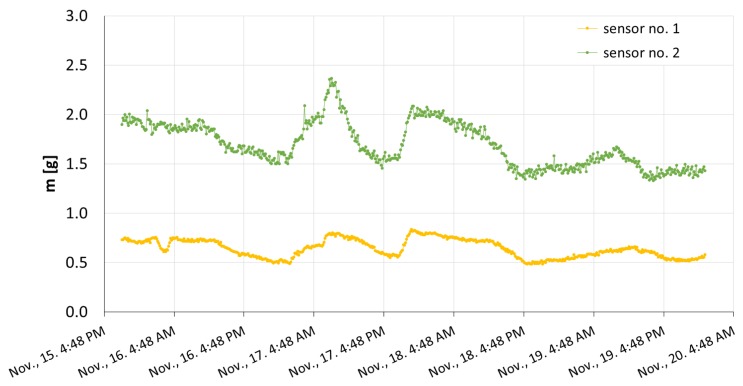
The amount of water, *m*, deposited within the ceramic plates of both sensors during field measurements.

The obtained outcomes were transformed according to the former calibration of the devices, which was described in Section 3.1. The measured TDR time was converted to the amount of water present in the porous plate. The final results are presented in Figure 6.

The maximal masses of water present within the plates were equal to 0.83 g for Sensor 1 and 2.37 g for Sensor 2. The respective ceramic plates were thus filled with water at the levels of 13% and 15% at the most, in relation to the saturation state. The above mass values corresponded to 1.5 × 10^−2^ and 4.3 × 10^−2^ g·cm^−2^, that is 0.15 and 0.43 mm H_2_O of atmospheric water deposition. The amount of water deposited within the porous plate of Sensor 2 was bigger in comparison to the plate of Sensor 1 throughout the whole experiment, because of the difference in plate volumes. This observation indicated that the volume of the functional element was crucial for the sensor readings.

The sensitivity of the particular device was defined as a ratio between the mass of water deposited within the ceramic plate, *m*, and the measured TDR time, *t_TDR_*. It was determined for both sensors in the ranges covering the water amount measured during field measurements, *i.e.*, for mass values not exceeding 1.0 and 2.5 g for Sensors 1 and 2, respectively. The sensitivity was calculated on the basis of calibration data discussed in Section 3.1 as a reciprocal of the slope of a linear fit performed within an appropriate mass range. The sensitivities of Sensors 1 and 2 were equal to 3.2 × 10^−3^ and 7.5 × 10^−3^ g·ps^−1^, respectively. The obtained values indicated that Sensor 1, whose volume is smaller, is more sensitive to the amount of water present in its functional element than Sensor 2.

The maximum absolute error of the TDR time readings performed by the employed TDR meter could be estimated as equal to 20 ps. Within the measurement range of the outdoor experiment, it corresponded to 6.4 × 10^−2^ and 1.5 × 10^−1^ g of the water present within the ceramic plates of Sensors 1 and 2, respectively, *i.e.*, to the atmospheric water deposition of 1.1 × 10^−4^ and 2.7 × 10^−3^ g·cm^−2^ or 1.1 × 10^−3^ and 2.7 × 10^−2^ mm H_2_O. The measurement resolution was equal to 1 ps, which gave the mass of water equal to 3.2 × 10^−3^ and 7.5 × 10^−3^ g for Sensors 1 and 2, respectively, estimated within the range set by field measurements. It corresponded to the atmospheric water deposition of 5.7 × 10^−5^ g·cm^−2^, i.e., 5.7 × 10^−4^ mm H_2_O for Sensor 1 and 1.3 × 10^−4^ g·cm^−2^, that is 1.3 × 10^−3^ mm H_2_O for Sensor 2.

### 3.3. Comparison with Soil

**Figure 7:**
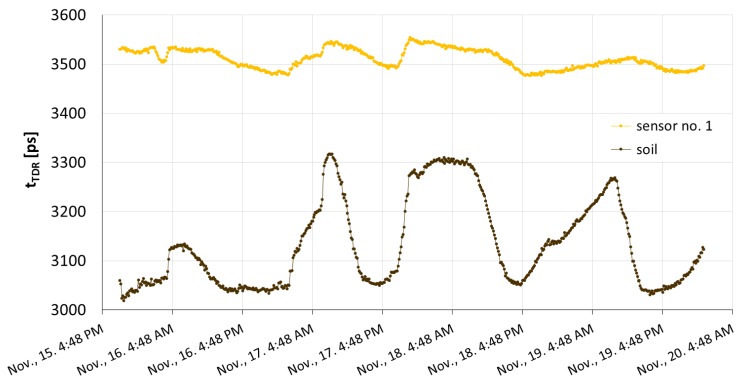
The TDR time, *t_TDR_*, for Sensor 1 and a soil sample of the same dimensions as its functional element recorded during field measurements.

The results of field measurements obtained with Sensor 1 were collated with the TDR atmospheric water deposition measurements in an actual soil sample. The comparison is depicted in Figure 7.

As may be inferred from Figure 7, both sensors revealed the same dynamics of water deposition within the porous materials, which were their functional elements, *i.e.*, the ceramic plate and the soil sample. Although the amount of water deposited within the plate was bigger than in the case of the investigated soil, the designed sensor was appropriate for detecting atmospheric water deposits and tracking the changes of the water amount within the porous materials, which appeared due to water deposition from the atmosphere.

The differences in water amounts measured by the two sensors resulted from the distinct structures of their functional elements, *i.e.*, ceramics and soil. Both chemical and physical properties determined the divergences of the obtained outcomes, as was described in detail in Section 2.3. For the above reasons, using the designed porous ceramic plate sensor in measurements of atmospheric water deposition within porous materials of various types would require material-specific calibration. The development of its methodology is planned as the topic of future research.

## 4. Conclusions

A porous ceramic plate sensor for the detection and tracking the variability of the intensity of atmospheric water deposits was designed, and two devices with different plate thicknesses were manufactured. The calibration of the probes was performed on the basis of the measurement data obtained at several ambient temperatures varying from 5 to 30 °C, and the field measurements were carried out. Additionally, the results obtained with one of the sensors and the outcomes obtained in an actual soil sample were compared.

The statement that the readings taken with the use of the porous ceramic plate sensors are practically independent of temperature was validated during the calibration phase. The temperature independence of the measurements was convenient, because it allowed obtaining a single calibration curve for the particular sensor. The curve served for converting the measured TDR time into the amount of water deposited in the porous material. The designed sensor was able to work at all temperatures, which admit the existence of liquid water under atmospheric pressure.

The readings obtained with the use of both porous ceramic plate sensors during field measurements showed that the construction of the device and the TDR technique were adequate for detecting and tracing the dynamics of atmospheric water deposition. On the basis of the conducted analyses, we noted that there existed an equilibrium state of the porous medium-atmosphere system. It can be observed in Figures 5 and 7 as a minimal value of TDR time achieved during the outdoor experiment. It was not consistent with the TDR time value for a completely dry porous plate measured in the laboratory. As may be inferred from Figure 6, the state corresponded to approximately 0.5 and 1.5 g of water within the plates of Sensors 1 and 2, respectively. Moreover, the amount of water present within the plates of the two sensors differed despite the same area of their top surfaces. It was bigger for the plate of a bigger volume, and hence, it can be stated that the water uptake was related to the volume of the plate. Thus, on the basis of our research, we may ascertain that the source of the measured water was not rainfall, which is constant per unit area, but atmospheric water deposition, which depends on the volume of the porous material.

The upper limits of the atmospheric water deposition measurement ranges of porous ceramic plate Sensors 1 and 2 were equal to 1.2 and 2.8 mm H_2_O, respectively. The respective sensitivities of Sensors 1 and 2 were equal to 3.2 × 10^−3^ and 7.5 × 10^−3^ g·ps^−1^. Since the material and internal structure of the functional element were the same in both cases, it may be inferred that the sensitivity depended on the volume of the plate. The sensor with a smaller volume had greater sensitivity, but the upper limit of its measurement range was smaller.

The comparative analysis of the porous ceramic plate sensor and the open container sensor filled with soil revealed that the outcomes obtained with the former device reflected the tendencies in the readings taken by the latter. This means that the designed sensor was appropriate for measuring water additions to the porous medium originating from atmospheric water deposition. Additionally, the structure of the ceramic plate was more stable and had better mechanical properties, as well as negligible susceptibility to the influence of temperature when compared with soil [37]. The material-specific calibration would be necessary for appropriate measurements of atmospheric water deposition in various porous materials. The elaboration of its methodology is planned as part of future research concerning the presented ceramic plate sensor.

The main advantages of the presented sensor over previously proposed devices for atmospheric water deposition measurements are the following. The innovative idea introduced to the construction of the device was the use of a porous material as its functional element. The porosity of the medium ensured that the phenomenon of direct water adsorption on the phase boundary was taken into account accurately. The response of the device was prompt, because water was absorbed uniformly in the bulk of the material. In comparison to previously employed non-porous condensation surfaces, the dependence of the measurement results on environmental conditions was reduced, and the readings could be taken frequently. Both the sensor and the TDR/MUX measuring unit are small and, hence, convenient to use. This is a considerable advantage, especially when compared to lysimetric devices, whose installation requires greater effort.

The employed TDR technique guaranteed the sufficient accuracy and resolution of measurements. The adequate sensitivity and accuracy were required for proper detection, because the intensity of water deposition is very low. Within the measurement range of the outdoor experiment, the error was estimated as equal to the atmospheric water deposition of 1.1× 10^−3^ and 2.7× 10^−2^ mm H_2_O for Sensors 1 and 2, respectively. High resolution was essential for the conducted experiments, because it allowed investigating the water deposition dynamics on the basis of changes observed during short time periods. The resolution of the readings, estimated within the range covered by field measurements for Sensors 1 and 2, was equal to the atmospheric water deposition of 5.7× 10^−4^ and 1.3× 10^−3^ mm H_2_O, respectively. The TDR/MUX measuring module allowed taking readings automatically at frequent time intervals. The TDR measuring system was equipped with a wireless connection, so it also enabled collecting the obtained data remotely. The measured physical quantity, *i.e.*, the TDR time, depended only on the amount of water present in the functional element of the sensor. This made the sensor insensitive to impurities, which could disturb the outcomes obtained with the use of other devices.

The potential range of applications of the developed sensor, whose construction and performance were presented in the paper, is wide and diversified. Apart from purely informative data, the acquisition and interpretation of which was described above, it could be potentially utilized as a supplementary device in meteorological stations or in sensor systems used in sustainable agriculture and forestry for determining optimal doses of water used for the irrigation of crops [38,41,42]. The proposed sensor could extend the functionality of agroclimatic measuring stations which use the TDR technique, because it can be attached instead of the conventional TDR probe, which measures the soil moisture. Moreover, it could serve for reference purposes in controlling desirable [43] or undesirable [44] water deposition and condensation on diverse natural surfaces and for monitoring the moisture of monuments and building construction elements. It could be also used for monitoring fire hazards in forests by measuring the litter moisture, for calibrating and validating the Soil Moisture and Ocean Salinity Satellite images, as well as to improve the accuracy of soil water balance models.

## Figures and Tables

**Table 1:** The percentage particle size distribution (PSD) of the examined soil.

**Particle Sizes (µm)**	0.01–2	2–20	20–50	50–100	100–250	250–500	500–1000	1000–2000

**PSD (%)**	1.91	11.17	10.96	6.46	13.64	28.18	23.47	4.21

**Table 2:** The values of the root mean square error (RMSE) of the experimental data for examined temperatures varying from 5 to 30 °C and the whole temperature range for the calibration curves of the porous ceramic plate sensors.

**Sensor No.**	**RMSE_5_**	**RMSE_10_**	**RMSE_15_**	**RMSE_20_**	**RMSE_25_**	**RMSE_30_**	**RMSE_5-30_**
1	0.44	0.56	0.70	0.55	0.61	0.57	0.58

2	0.41	0.30	0.63	0.44	0.34	0.61	0.40
